# Two new cases of anti-Ca (anti-ARHGAP26/GRAF) autoantibody-associated cerebellar ataxia

**DOI:** 10.1186/1742-2094-10-7

**Published:** 2013-01-15

**Authors:** Sven Jarius, Pedro Martínez-García, Adelaida León Hernandez, Jan Christoph Brase, Kathrin Borowski, Jens Ulrich Regula, Hans Michael Meinck, Winfried Stöcker, Brigitte Wildemann, Klaus-Peter Wandinger

**Affiliations:** 1 Division of Molecular Neuroimmunology, Department of Neurology, University of Heidelberg, Im Neuenheimer Feld 400, Heidelberg, 69120, Germany; 2 Immunology Service, University Hospital Virgen de la Arrixaca, El Palmar, Murcia, 30120, Spain; 3 Radiodiagnostic Service, University Hospital Virgen de la Arrixaca, El Palmar, Murcia, 30120, Spain; 4 Unit Cancer Genome Research, Division of Molecular Genetics, German Cancer Research Center and National Center of Tumor Diseases, Im Neuenheimer Feld 460, Heidelberg, 69120, Germany; 5 Current address: Sividon Diagnostics GmbH, Nattermannallee 1, Cologne, 50829, Germany; 6 Institute for Experimental Immunology, affiliated to Euroimmun, Seekamp 31, Luebeck, 23560, Germany; 7 Division of Neurophysiology, Department of Neurology, University of Heidelberg, Im Neuenheimer Feld 326, Heidelberg, 69124, Germany; 8 Institute for Neuroimmunology and Clinical MS Research, Center for Molecular Neurobiology Hamburg (ZMNH), University Medical Center Eppendorf, Falkenried 94, Hamburg, 20251, Germany

**Keywords:** Autoimmune cerebellar ataxia, Purkinje cells, Autoimmunity, Autoantibodies, RhoGTPase-activating protein 26 (ARHGAP26), GTPase regulator associated with focal adhesion kinase pp125 (GRAF), Oligophrenin-1-like protein, Paraneoplastic, Ovarian cancer

## Abstract

Recently, we discovered a novel serum and cerebrospinal fluid (CSF) autoantibody (anti-Ca) to Purkinje cells in a patient with autoimmune cerebellar ataxia (ACA) and identified the RhoGTPase-activating protein 26 (ARHGAP26; alternative designations include GTPase regulator associated with focal adhesion kinase pp125, GRAF, and oligophrenin-1-like protein, OPHN1L) as the target antigen. Here, we report on two new cases of ARHGAP26 autoantibody-positive ACA that were first diagnosed after publication of the index case study. While the index patient developed ACA following an episode of respiratory infection with still no evidence for malignancy 52 months after onset, neurological symptoms heralded ovarian cancer in one of the patients described here. Our finding of anti-Ca/anti-ARHGAP26 antibodies in two additional patients supports a role of autoimmunity against ARHGAP26 in the pathogenesis of ACA. Moreover, the finding of ovarian cancer in one of our patients suggests that anti-Ca/anti-ARHGAP26-positive ACA might be of paraneoplastic aetiology in some cases. In conclusion, testing for anti-Ca/anti-ARHGAP26 should be included in the diagnostic work-up of patients with ACA, and an underlying tumour should be considered in patients presenting with anti-Ca/ARHGAP26 antibody-positive ACA.

## Introduction

We recently described a novel serum and cerebrospinal fluid (CSF) autoantibody in a patient with subacute autoimmune cerebellar ataxia (ACA) [1]. In addition, we demonstrated that this antibody (termed anti-Ca), which selectively binds to Purkinje cells when incubated with primate or murine cerebellum tissue sections, targets the RhoGTPase-activating protein 26 (ARHGAP26; alternative designations include GTPase regulator associated with focal adhesion kinase pp125, GRAF, and oligophrenin-1-like protein, OPHN1L).

Here we report on two new cases of ACA with anti-Ca/anti-ARHGAP26 antibodies that were diagnosed since our first publication on this novel serum reactivity. While the index patient had developed ACA following an episode of respiratory infection with still no evidence for cancer 52 months after onset, ACA heralded carcinoma in one of the patients described here, suggesting that anti-Ca/anti-ARHGAP26 is a potential marker of paraneoplastic ACA.

## Case reports

### Case 1

**Figure 1:**
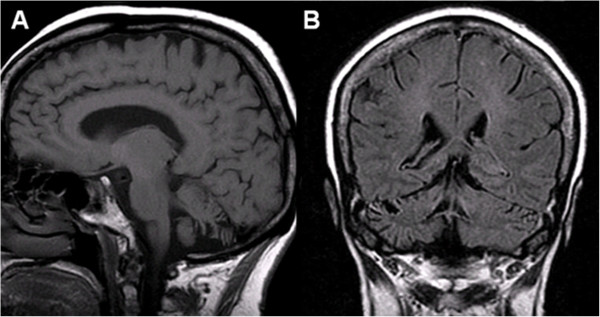
**Magnetic resonance imaging of the brain, patient 1.** Sagittal (**A**, T1 weighted) and coronal (**B**, FLAIR T2 weighted) MRI showing cortical atrophy in the cerebellar hemispheres and the inferior vermis with enlargement of the cerebellar sulci, the fourth ventricle and the inferior cerebellar cistern, but no atrophy of the cerebral cortex, midbrain, or pons.

This previously healthy 68-year-old Caucasian woman presented to her general practitioner with a three-month history of dizziness. A cranial MRI was rated normal at that time except for an empty sella. Laboratory analysis disclosed asymptomatic hyperprolactinemia. The patient’s medical history was otherwise unremarkable. No infections were reported to have preceded the onset of symptoms and the family history was negative for neurological and oncological diseases. No specific treatment was ordered at that time. Four months later, the patient was admitted to hospital with signs of ataxia. Neurological examination demonstrated a multidirectional gaze-evoked nystagmus, cerebellar dysarthria, atactic gait, and severe difficulties in standing with feet together with eyes open; tandem walking was impossible. Deep tendon reflexes and plantar responses were normal. No signs of meningeal irritation were found. No apparent cognitive deficits were noted. At that time, MRI of the head showed mild isolated cerebellar atrophy. Visual and somotosensory-evoked potentials were normal. Serum analysis revealed anti-neuronal antibodies of then unknown specificity and low-titre smooth muscle antibodies. Routine laboratory findings were normal except for a slightly elevated erythrocyte sedimentation rate (36 mm after 1 h) and increased lactate dehydrogenase (362 U/l; reference range, <250) blood levels. No CSF analysis was performed at that time. A CT scan of the abdomen and pelvis disclosed enlarged retroperitoneal and mediastinal lymph nodes. Findings from mammography and from X-ray and CT scanning of the thorax were normal. Histology of the abdominal mass demonstrated a nondifferentiated carcinoma likely of gynaecological origin. Laboratory analysis showed elevated serum levels of cancer antigen (CA) 125 (2198 IU/ml; reference range, <35), CA15-3 (54 IU/ml; reference range, <25), and neuron-specific enolase (NSE; 39 ng/ml; reference range, <16.3). Ovarian carcinoma was suspected and treatment with carboplatin and docetaxel was commenced (seven cycles over a period of six months). After the third cycle, treatment with rituximab (four cycles) and intravenous immunoglobulins (two cycles over five days each) was added, which resulted in partial improvement of the patient’s neurological symptoms. Eight months later, follow-up examinations revealed a new retroperitoneal mass (6 x 5 cm) and a nodule on the left ovarian vein; histology suggested lymphatic infiltration due to a carcinoma likely of gynaecological origin, and double adnexectomy and hysterectomy were performed. At last admission, 24 months after onset, the patient reported increased gait instability, nausea, and vomiting. Brain MRI revealed atrophy of the cerebellar hemispheres and the cerebellar vermis (Figure 1). Despite second-line chemotherapy with carboplatin and cyclophosphamide, her neurological symptoms are still advancing slowly. 

### Case 2

A 38-year-old Iranian man presented with a nine-month history of cerebellar ataxia and dysarthria, weight loss, and vomiting. Brain MRI showed cerebellar atrophy. CSF analysis showed CSF-restricted oligoclonal bands and 5 cells/μl. No further clinical data were available from this patient retrospectively.

## Immunological studies

### Methods

#### Immunohistochemistry (IHC)

IHC was performed on cryosections of adult rhesus monkey, rat, and mouse cerebellum tissue (Euroimmun, Luebeck, Germany) as previously described [1]. Briefly, sections were pretreated with formalin and 1% 3-[(3-cholamidopropyl)dimethylammonio]-1-propanesulfonate (CHAPS) in PBS, blocked with 10% goat serum and then incubated with patient serum for 1 h. Binding of human immunoglobulin (Ig)G, IgA, and IgM to CNS tissue was detected by using polyclonal goat anti-human IgG antibodies conjugated to fluorescein isothiocyanate (FITC) (Euroimmun) or Alexa Fluor™ (AF) 568 (Invitrogen, Karlsruhe, Germany), and polyclonal goat anti-human IgM and anti-human IgA antibodies conjugated to FITC (Euroimmun), respectively. Sections were then mounted using glycerol standard immunofluorescence mounting medium containing 4',6-diamidino-2-phenylindole (DAPI) (1:1000) (Euroimmun) or ProLong Gold antifade reagent (Invitrogen). Slides were analyzed on a Nikon 90i upright fluorescence microscope and a Nikon A1 confocal microscope (Nikon Imaging Center, University of Heidelberg, Heidelberg, Germany).

#### Immunoglobulin G (IgG) subclass analysis

To evaluate IgG subclasses, serum and CSF samples were tested by IHC on mouse cerebellum sections as described above, with the following modifications: sections were blocked with 10% donkey serum; nonconjugated sheep anti-human IgG antibodies specific for IgG subclasses 1 to 4 (Binding site, Germany) were substituted for the FITC-labeled goat anti-human IgG antibody; and AF568-labeled donkey anti-sheep IgG (Invitrogen; absorbed against human IgG) was used to detect the subclass-specific antibodies.

#### Preadsorption experiments

To confirm ARHGAP26 specificity, sera were preadsorbed overnight with human full-length ARHGAP26 (Biozol, Eching, Germany) and supernatants were tested by IHC as described above.

#### Dot blot assay

Protran BA79 nitrocellulose membranes (0.1 μm) (Whatman, Fisher Scientific, Schwerte, Germany) were spotted with a 0.14 μg/μl solution of human full-length ARHGAP26 (10 μl/spot; Biozol). After drying, membranes were blocked with 5% bovine serum albumin (BSA) in Tris-buffered saline (TBS) for 1 h at room temperature (RT), washed three times in TBS with 0.05% Tween (TBS-T), and then incubated with a 1:20 dilution of the patient's serum in 0.1% BSA/TBS-T for 1 h at RT. A donkey anti-human IgG antibody labeled with IRdye 700DX (Rockland, Gilbertsville, PA, USA) was used to detect bound IgG. Stripes were finally washed in TBS and analyzed using an Odyssey™ fluorescence scanner (Licor, Lincoln, NE, USA) and Odyssey™ 2.0.40 application software (Licor).

#### Testing for coexisting antibodies

All samples were tested for anti-Hu, -Yo, -Ri, -CV2/CMRP5, -Ma/Ta, -Tr, -amphiphysin, -aquaporin-4, -GAD, -MAG, and -myelin antibodies by IHC on mouse, rat, and monkey brain and peripheral nerve tissue sections (Euroimmun, Luebeck, Germany), for anti-Hu, -Yo, -Ri, -CV2/CMRP5, -Ma/Ta, and -amphiphysin antibodies by using a commercially available line blot assay (Euroimmun, Germany), and for anti-NMDA-type glutamate receptor, anti-AMPA-type glutamate receptor, anti-GABA-B receptor, anti-CASPR2, anti-LGI1, anti-AQP4, and anti-glycine receptor antibodies using a panel of recombinant cell-based assays (Euroimmun, Germany).

## Results

**Figure 2:**
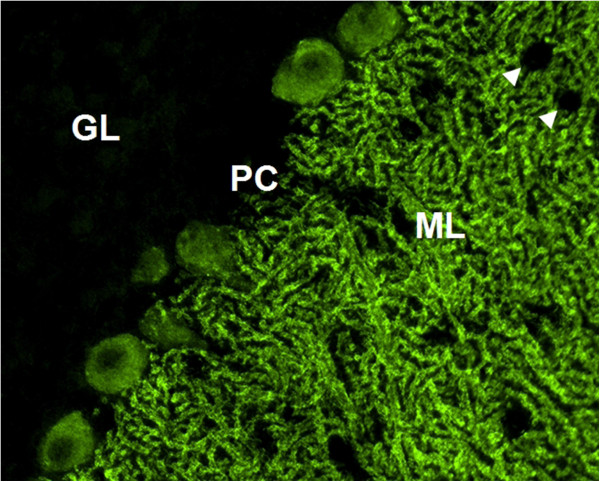
**Indirect immunofluorescence (IIF) on mouse cerebellum tissue sections revealed the typical anti-Ca staining pattern as previously described**[1]**.** GL, granular layer; PC, Purkinje cell layer; ML, molecular layer. Interneurons are spared (arrowheads).

**Figure 3:**
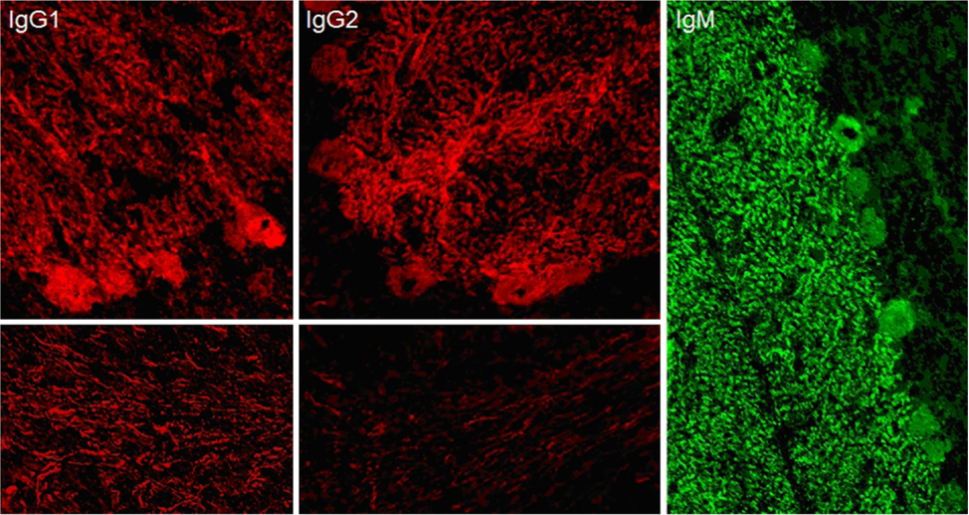
**Anti-Ca antibodies belonged mainly to the IgG1 and IgG2 subclasses (left and middle panels).** Patient 1 was, in addition, positive for anti-Ca antibodies of the IgM class (right panel) and patient 2 for anti-Ca antibodies of the IgA class (not shown).

**Figure 4:**
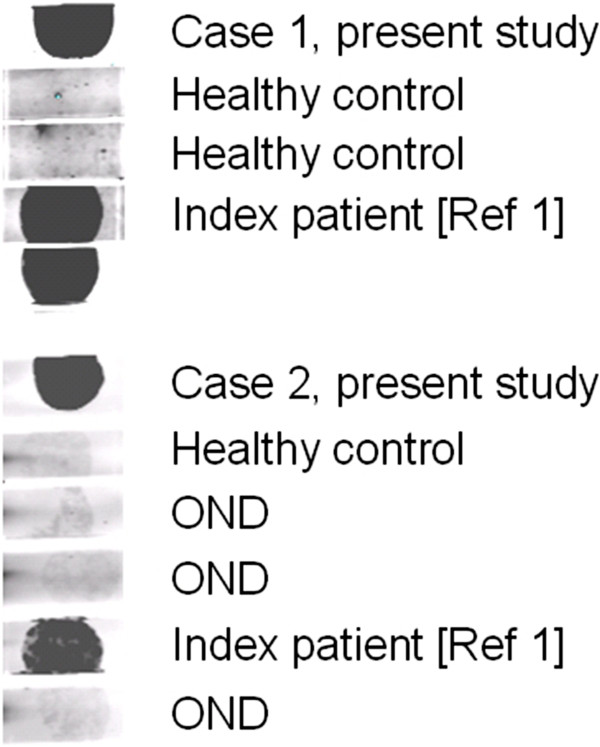
**Binding of serum IgG to human recombinant full-length ARHGAP26 as demonstrated in a dot blot assay.** OND, other neurological diseases.

**Figure 5:**
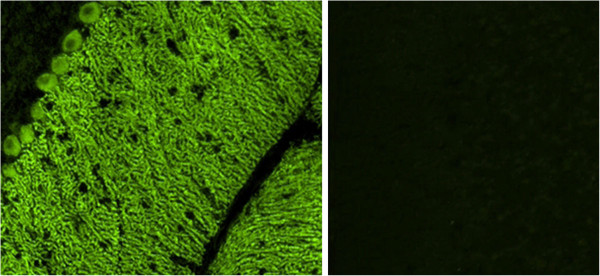
Preadsorption of the patients’ sera with recombinant human full-length ARHGAP26 but not preadsorption with a control protein resulted in complete disappearance of the typical anti-Ca cerebellar staining pattern as detected by IIF (left panel: binding of serum IgG from patient 1 to a cerebellum tissue section before preadsorption with ARHGAP26, right panel: after preadsorption with ARHGAP26).

HC on formalin-fixed cerebellum tissue sections revealed selective binding of IgG to somata, axons, dendritic trunks, and dendritic branches of Purkinje cells (PCs) in a pattern identical to that recently described in a patient with ARHGAP26 antibodies (anti-Ca) and ACA (Figure 2) at a titre of 1:32,000 in patient 1 and 1:3,200 in patient 2. As in the index patient, anti-Ca antibodies belonged mainly to the IgG1 subclass (patient 1: IgG1>=IgG2>>IgG4>>IgG3; patient 2: IgG1>>IgG2>IgG3). In addition, patient 1 was positive for anti-Ca antibodies of the IgM class (Figure 3) and patient 2 for anti-Ca antibodies of the IgA class. Both patients tested positive in a dot blot assay using recombinant human full-length ARHGAP26 as antigen as previously described (Figure 4). Preadsorption of the patient’s serum with recombinant human full-length ARHGAP26, but not preadsorption with a control protein, resulted in complete disappearance of the cerebellar staining pattern as detected by IHC, again confirming the antibodies’ specificity to ARHGAP26 (Figure 5). Using IHC on brain tissue sections, a commercial line blot assay, and a panel of antigen-specific cell-based assays to detect classical paraneoplastic antibodies, no evidence was found for anti-Hu, anti-Ri, anti-Yo, anti-Ma, anti-Ta, anti-Tr, PCA-2, ANNA-3, anti-CV2/CRMP5, anti-amphiphysin, ANNA3, PCA2, anti-GAD, anti-NMDA receptor, anti-AMPA receptor, anti-GABA-B receptor, anti-CASPR2, anti-LGI1, anti-glycine receptor, anti-myelin, or anti-MAG antibodies. All diagnostic tests reported here were performed as part of the patients' routine clinical workup. 

## Discussion

Recently, we reported on a newly discovered serum and CSF autoantibody in a patient with ACA [1]. This new antibody bound selectively to Purkinje cell somata, dendrites, and axons on primate and murine cerebellum tissue sections, and was shown to target ARHGAP26. Here, we report on two newly diagnosed cases of anti-Ca/anti-ARHGAP26-positive ACA.

Occurrence of ARHGAP26 antibody-positive ACA led to the diagnosis of ovarian carcinoma in one of the patients reported here, suggesting a possible paraneoplastic aetiology of the condition. Paraneoplastic neurological disorders count among the most common causes of antibody-associated ACA [2,3]. It is of note in this context that ARHGAP26 has been shown to be expressed in a subset of ovarian cancer tissues, partly at high levels, while it is absent or present only at low levels in normal ovarian tissue [4]; however, no tumour tissue from patient 1 was available for analysis in this study.

Antibodies previously demonstrated in patients with paraneoplastic ACA included anti-Hu [5], anti-Yo [6], anti-CV2/CRMP5 [7,8], anti-Tr [9,10], anti-Zic4 [11], anti-protein kinase C gamma (PKCγ) [12], anti-mGluR1 [13,14], anti-PCA2 [15], anti-ANNA3 [16], or voltage-gated calcium channels (VGCC) [17]. None of these antibodies was detected in the patient reported here.

Histologically, a diagnosis of undifferentiated carcinoma was made. However, as a caveat, elevated serum levels of neuron-specific enolase (NSE), a marker of neuroendocrine tumours, were detected. This is of potential interest since tumours of neuroendocrine differentiation such as small-cell lung cancer and neuroblastoma have previously been implicated in a wide range of paraneoplastic neurological disorders, including ACA [2,18]. As no secondary carcinoma of neuroendocrine differentiation has been found in repeated follow-up examinations, we cannot exclude that the primary tumour contained neuroendocrine components that went unrecognized. Notably, ARHGAP26 has been found to be upregulated in neuroendocrine tumours [19]. Alternatively, the elevated NSE serum levels might be of neuronal origin, reflecting the marked neuronal loss as detected on MRI.

In the second case reported here, the tumour status is unknown as the patient is lost to follow-up; however, the development of ACA was reportedly associated with unusual weight loss in this patient.

Now that it is clear that anti-ARHGAP26/GRAF is present in more patients with ACA, studies on the immunopathological impact of this new serum reactivity are warranted. So far, it is unknown whether the antibody itself causes neurological damage (as has been shown for some of the novel anti-CNS autoantibodies described over the past of couple of years [20]) or whether the antibody is merely a disease marker of ACA while the actual damage is T cell-mediated (as it is thought to be the case with the classical onco-neuronal antibodies). Of note, as in the index case, anti-Ca/anti-ARHGAP26 belonged to the complement-activating IgG1 subclass in the two new cases reported here, confirming that these new antibodies may possibly act on PCs via complement-dependent mechanisms. In this context, it is of note that patient 1 was, in addition to IgG, positive for IgM antibodies to ARHGAP26. IgM antibodies are generally known to be more potent activators of complement than IgG. Autoantibodies of the IgM class have been reported also in other autoimmune diseases of the CNS [21]. By contrast, patient 2 as well as well as the index patient [1] were negative for ARHGAP26-IgM.

Our finding of high-titre anti-Ca/anti-ARHGAP26 antibodies in two additional patients with ACA strongly supports a role for autoimmunity against ARHGAP26 in the pathogenesis of this rare condition and proves that the index patient was not a singular case. Moreover, the finding of ovarian cancer in one of our patients suggests that anti-Ca/anti-ARHGAP26-positive ACA might be of paraneoplastic aetiology in some cases. In conclusion, testing for anti-Ca/anti-ARHGAP26 should be included in the diagnostic work-up of patients with ACA; while more cases have to be evaluated before a strict recommendation can be made as to whether broad tumour screening is generally required in patients with anti-Ca/ARHGAP26 antibody-positive ACA, non-harmful screening procedures such as ultrasound examination for ovarian cancer and, possibly, tumour marker testing seem warranted.

## Competing interests

The work of BW was supported by research grants from Merck Serono and Bayer Healthcare. KPW, KB and WS are employees of Euroimmun, Luebeck, Germany. Euroimmun had no role in study design, preparation of the manuscript, or decision to publish.

## Authors’ contributions

SJ, KPW and BW conceived and designed the study. PMG and ALH performed the clinical examinations and analyzed the MRI data. SJ, PMG, KPW, KB, JCB, and WS were involved in performing and/or analyzing the experiments. SJ drafted the manuscript. All authors participated in the critical revision of the manuscript for important intellectual content; and all authors have given final approval of the version to be published.

## Authors’ information

Senior authors: Brigitte Wildemann and Klaus-Peter Wandinger.
